# Involvement of Reduced Microbial Diversity in Inflammatory Bowel Disease

**DOI:** 10.1155/2016/6951091

**Published:** 2016-12-15

**Authors:** Dawei Gong, Xiaojie Gong, Lili Wang, Xinjuan Yu, Quanjiang Dong

**Affiliations:** ^1^Department of Central Laboratories and Gastroenterology, Qingdao Municipal Hospital, School of Medicine, Qingdao University, Qingdao 266071, China; ^2^Department of Emergency Surgery, The Fifth People's Hospital of Ji'nan, Ji'nan 250022, China

## Abstract

A considerable number of studies have been conducted to study the microbial profiles in inflammatory conditions. A common phenomenon in inflammatory bowel disease (IBD) is the reduction of the diversity of microbiota, which demonstrates that microbial diversity negatively correlates with disease severity in IBD. Increased microbial diversity is known to occur in disease remission. Species diversity plays an important role in maintaining the stability of the intestinal ecosystem as well as normal ecological function. A reduction in microbial diversity corresponds to a decrease in the stability of the ecosystem and can impair ecological function. Fecal microbiota transplantation (FMT), probiotics, and prebiotics, which aim to modulate the microbiota and restore its normal diversity, have been shown to be clinically efficacious. In this study, we hypothesized that a reduction in microbial diversity could play a role in the development of IBD.

## 1. Introduction

Inflammatory bowel disease (IBD) is a chronic disease, and the two main forms of IBD are ulcerative colitis (UC) and Crohn's disease (CD). IBD has been shown to be associated with increased morbidity in developed countries and developing countries that are gradually adopting a more modern lifestyle. IBD has been reported to be caused by a multitude of factors such as genetics, environment, immune system, and gut microbiota. Although its exact pathogenesis remains unclear, dysbiosis of the microbiota in the intestinal tract is widely accepted to initiate or promote intestinal inflammation [1–3].

An increasing number of studies have used noncultured* 16S* rRNA sequencing technology to reveal intestinal microbial profiles in IBD and healthy controls. Some meaningful features were found. The gastrointestinal tract contains several hundred microbial species [4–7], most of which belong to the Firmicutes, Bacteroidetes, Proteobacteria, and Actinobacteria phyla [8, 9]. A balanced microbial population in the human gut could benefit the human body by providing nutrients, maintaining immune homeostasis, and granting niche protection. However, recent studies have demonstrated a dysbiosis in IBD patients. CD patients had significantly higher populations of* Streptococcus* and* Enterococcus* and lower populations of* Coprococcus*,* Roseburia*,* Faecalibacterium*, and* Ruminococcus* compared with healthy controls [10]. Furthermore, the abundance of* Bacteroides*,* Enterococcus*,* Blautia*, and* Escherichia-Shigella* genera was significantly increased in patients with UC, and the abundance of* Coprococcus* decreased compared with healthy controls [10]. Andoh et al. found that the abundance of* Clostridium* was decreased in patients with active UC and inactive/active CD patients, whereas the abundance of* Bacteroides* significantly increased in the patients with CD [11]. Although the altered microbial profiles did not show any consistent results across many studies, a common feature, that is, reduced microbiota diversity, emerged in all patients with IBD [7, 12–16].

The diversity of microbiota is closely associated with disease conditions. In a study on microbiota signatures in eczema, Nylund et al. found that the severity of eczema was inversely correlated with the microbial diversity (*r* = −0.54; *P* = 0.005), indicating that the lower the microbial diversity, the higher the severity of eczema [17]. Additionally, the microbial diversity increased with improvement of symptoms in eczema [17]. A similar relationship was found between the microbial diversity and disease severity in human IBD [18]. Russell et al. treated neonatal mice with vancomycin and reported that this resulted in a reduction in microbial diversity accompanied by an increase in the severity of asthma [19]. The level of diversity is also related to the response to therapy in patients. For example, children with UC who responded to corticosteroid medication were reported to have a higher diversity than nonresponders [20].

Little attention has been paid to the role of reduced microbial diversity in the function of the intestinal ecosystem. This review intends to reveal the relationship between a reduction in diversity and IBD from a biological perspective.

## 2. Reduced Microbial Diversity in IBD

A previous study compared the intestinal microbial flora between patients with active CD and healthy controls using* 16S* rRNA gene sequencing. The mean Shannon diversity was lower in CD patients than in the healthy controls [21]. Similar to these findings, in a multicenter study, fecal samples collected from 161 patients with CD and 121 healthy individuals found that the active/inactive CD patients had a significantly lower Shannon diversity than the healthy controls [22]. Sha et al. analyzed the diversity of fecal microbiota in IBD patients compared with healthy controls using denaturing gradient gel electrophoresis. The participants included patients with UC, patients with CD, and healthy controls. The microbial diversity was remarkably lower in IBD patients than in the healthy control group, and the reduced microbial diversity was more obvious in the active UC and active CD groups [13]. Additionally, studies of microbial profiles from UC patients revealed that these patients have lower fecal microbial diversity and lower abundance of major anaerobic bacteria (*Bacteroides* and* Clostridium* subcluster XIVa) compared with healthy controls [23]. In another study, microarray hybridization was used to study the characteristics of the microbiota from healthy controls and children hospitalized with severe UC. The richness, evenness, and biodiversity of the gut microbiota were all significantly reduced in children with UC compared with healthy controls; furthermore, a reduction in the abundance of* Clostridium* and an increase in the class Gammaproteobacteria were found [20].

The precise causal relationship between the inflammatory state and a reduction in bacterial diversity remains unknown. To determine whether defects in the mucosal barrier and bacterial dysbiosis are inherently abrogated in the terminal ileum (TI) of patients with UC (where inflammation is absent), the TI was biopsied from patients with CD and UC and from healthy controls without IBD [24]. Despite the absence of ileitis, UC patients displayed ileal barrier depletion and a reduction in the *α*-diversity compared with pediatric patients without IBD [24]. In a study on mice, when antibiotics were used to deplete the gut microbiota, a reduction in diversity and mild gut inflammation occurred [25]. In another study, microbial profiles of patients with CD, their healthy siblings, and unrelated healthy individuals were sequenced and analyzed. Healthy siblings who were at a higher risk of developing CD had lower core microbial diversity than low-risk healthy controls (although the microbial diversity in the siblings was higher than that in the CD patients), suggesting that the loss of core microbial diversity may be a fundamental step in the pathogenesis of CD [26]. Interestingly, the hygiene hypothesis suggests that improved hygiene conditions may lead to a reduction in the intestinal microbial diversity and that it may in turn be responsible for the development of IBD [27].

## 3. Attempt to Restore Normal Diversity to Alleviate IBD

FMT involves the transfer of fecal suspension from a healthy donor to the intestinal tract of a recipient, modulating imbalanced gut microbiota and restoring normal diversity and bacterial composition of the intestine. FMT therapy is currently used in treatment of intestinal inflammation [28].

FMT has received extended attention in the treatment of IBD [29–31]. Many studies have revealed that FMT can induce the remission of some IBD patients. Moayyedi et al. conducted FMT in patients with active UC in a randomized controlled trial. In their study, patients with UC were examined using flexible sigmoidoscopy at the start of the study and were randomly assigned to groups that received an enema of either 50 mL of FMT (from healthy anonymous donors, *n* = 38) or 50 mL of placebo (water, *n* = 37) once per week for six weeks. The primary outcome was remission of UC, defined as a Mayo score ≤2 with an endoscopic Mayo score of 0 at week 7 of treatment. In their study, nine of the patients who received FMT (24%) and 2 who received placebo (5%) achieved remission (*P* = 0.03) [32]. Additionally, the fecal samples of patients receiving FMT had greater microbial diversity compared with baseline [33]. A systematic review and meta-analysis from a large multigroup sample after FMT therapy found that remission after FMT could reach 36.2% [34]. A subgroup analysis revealed that the clinical remission of the UC group and the remission rate of the CD group reached 22% and 60.5%, respectively [34]. FMT has also been reported to have the best efficacy in treating patients when first diagnosed [32].

*Clostridium difficile* infection (CDI) is caused by toxin-producing* C. difficile* and features a range of symptoms from mild diarrhea to potentially lethal conditions such as pseudomembranous colitis. Low et al. found that the microbial diversity was lower in patients with CDI than in healthy controls, and alterations in the composition included a reduction in the abundance of Firmicutes and Bacteroidetes as well as an increased abundance of Proteobacteria [35]. Notably, a study on CDI reported that the reduction in microbial diversity occurs prior to the occurrence of CDI, emphasizing the promoting effect of reduced diversity on intestinal inflammation [36]. Antibiotic therapy for CDI cannot achieve the desired effect and can induce recurrent* Clostridium difficile* infection (rCDI) [37]. In other studies, increased microbiota diversity could be achieved in recipients who achieved remission after FMT [35, 38, 39]. Accordingly, when FMT was used to treat CDI, a significant curative effect could be achieved [28, 40]. van Nood et al. studied the efficacy of FMT in rCDI compared with vancomycin to treat recurrent infection. They found a significantly higher rate in the resolution of* C. difficile*-associated diarrhea in the FMT group with the resolution of symptoms in nearly all patients in the FMT group [40]. After the FMT procedure, the recipients showed increased microbial diversity at levels similar to the microbial features of the healthy donor [40]. To investigate the eradication of* C. difficile* and changes in the microbiome following FMT in children with and without IBD, 8 children (5 with IBD and 3 without IBD) with a history of recurrent CDI (≥3 recurrences) received FMT via colonoscopy. All 8 children showed the resolution of CDI symptoms and eradication of* C. difficile* at 10–20 weeks and 6 months after the administration of FMT [41]. There was also an increase in the intestinal microbial diversity after FMT therapy [41]. Three systematic reviews and a meta-analysis on CDI treated with FMT showed an average curative rate of approximately 90% [42–44]. Furthermore, a controlled trial revealed that the curative rate of rCDI treated with FMT could reach 81% [40]. FMT therapy is associated with fewer adverse reactions [45, 46]. The most successful application of FMT was reported in the treatment of CDI [47]. Although the pathogenesis of CDI is significantly different from that of IBD, the microbiota of patients with both conditions have been found to be similar, and both conditions have shown successful outcomes following FMT treatment, especially in CDI, indicating that the normal intestinal microbiota plays an indispensable role in human homeostasis and that reduced diversity of the microbiota could cause intestinal inflammation.

## 4. Potential Factors Contributing to a Reduction in Microbial Diversity

The gastrointestinal tract environment may be considered as an ecosystem. It includes the mucosal epithelium, mucus layer, bacteria, viruses, funguses, parasites, and archaea [4]. The health of the gastrointestinal ecosystem is primarily represented by its microbial diversity. A balanced ecosystem represents normal ecological function and gut health. However, the ecological environment is disturbed in IBD, resulting in reduced microbial diversity. Therefore, the question arises: what are the factors that induce a reduction in microbial diversity? Knowing the exact mechanism could help reduce the risks associated with reduced diversity and provide therapeutic strategies to treat it.

### 4.1. Role of Antibiotics, Diet, and Environments

Antibiotics are widely used and can lead to significant changes in the composition of the intestinal microbial flora [48–51]. Jakobsson et al. studied the effects of metronidazole and clarithromycin on intestinal microbiota and found that the drugs caused a significant diversity reduction [52]. The antibiotics disrupted the microbiota and caused antibiotic-associated diarrhea [53, 54]. Diet is also responsible for altering the composition of the intestinal microbiota [55]. In a recent study, Sonnenburg et al. reported that a diet low in dietary fiber could decrease the diversity of intestinal microbiota in mice and that a diet that is persistently low in dietary fiber resulted in a progressive loss of microbial diversity in subsequent generations of mice. Conversely, a diet rich in dietary fiber helped maintain high intestinal microbiota diversity in mice of all generations [56]. This phenomenon may explain the higher morbidity of IBD in Western countries, which tend to have high-fat low-fiber diets. Recently, a systematic review focusing on the impact of diet on gut microbiota also indicated that a fiber-rich diet could elevate microbial diversity [57]. Environmental factors can also significantly impact the microbial profile [58]; the microbial profiles in Malawian and Venezuelan populations are more diverse than those of adults and children residing in the US [59].

### 4.2. Role of Genetics, Immune System, and Mucus

To study the potential role of the chromatin remodeler CHD1 in shaping the gut microbiome of* Drosophila melanogaster*, Sebald et al. performed deep* 16S* rRNA gene sequencing of gut microbiota from* Chd1*^−/−^ and control* Chd1*^*WT/WT*^ flies, which carried a wild-type* Chd1* rescue transgene in a Chd1-deficient genetic background. Interestingly, principal coordinate analysis revealed clear distinctions between the microbiota of mutant and wild-type flies. The* Chd1*^−/−^ flies had a significantly lower microbial diversity than the* Chd1*^*WT/WT*^ controls [60]. This implies the genetic function in shaping the structure and composition of microbiota. Lim et al. investigated the effect of heritability and host genetics on gut microbiota and metabolic syndrome and reported that the patients with metabolic syndrome had lower microbial diversity than healthy individuals. The microbial profiles were significantly associated with the special host genotype [61, 62]. The role of adaptive immunity on the gut microbiota was investigated in a mouse model to determine the role of immunity in the regulation of gut microflora. The microbiota of immunodeficient Rag1^−/−^ mice and wild-type mice housed in the same conditions were analyzed using* 16S* rRNA sequencing [63]. The results demonstrated that Rag1^−/−^ mice had distinct microbiota and had a higher increase in microbial diversity with increasing age compared to wild-type mice [63]. To a certain extent, this study may imply that immune enhancement could suppress microbial diversity. Furthermore, the human intestinal tract surface is covered with a mucus layer that prevents intimate interaction between the intestinal epithelium and bacteria [64, 65]. The colon mucus layer in humans consists of an inner layer and an outer layer. The viscosity of the outer layer is low, making it easy to penetrate by bacteria. However, the inner layer of the mucus is viscous and sterile [66]. The mucus plays an indispensable role in maintaining homeostasis in the human intestinal tract. During inflammation, the mucus layer is impaired and infiltrated by microbes [64].* MUC2*, which is primarily produced by goblet cells, is a major component of the colonic mucus layer [66, 67]. In a* MUC2*-deficient mouse model, mice lacking* MUC2* were found to develop spontaneous colitis [68, 69]. Mucin is an energy source for intestinal microbiota and could regulate the composition of intestinal microflora [70]. Bel et al. studied TMF^−/−^ mice lacking TMF/ARA160 and found that these mice produce thick and uniform colonic mucus. The microbial features of TMF^−/−^ knockout mice and wild-type mice were analyzed and the Shannon diversity index was higher in the former than in the latter group [71]. When dextran sulfate sodium (DSS) was used to induce colitis in two mice groups, the results showed that the knockout mice (with higher diversity) exhibited an attenuated response to DSS compared to their wild-type counterparts. Both groups of mice were cohoused for 4 weeks, and the diversity of microbiota was higher in the cohoused wild-type mice than in the wild-type mice housed alone; additionally, the cohoused wild-type mice had diminished susceptibility to induced colitis [71].

Many studies have already reported the association between these factors and IBD. Interestingly, these factors, which contribute a reduction in microbial diversity, are also risk factors for developing IBD [72–76].

## 5. Mechanism of Reduction in Diversity

Biodiversity plays an important role in maintaining a balanced ecosystem by contributing to the stability of an ecosystem as well as ecological functioning [77–79]. A high diversity provides the ecosystem with strong stability, which is defined as the ability to resist disturbance and to maintain normal ecological function [80]. When diversity is lost, the stability of an ecosystem decreases, which means that it is more susceptible to even minor assaults. This may explain why the lower gut microbial diversity observed in Western populations is associated with a higher morbidity rate of IBD. Consequently, IBD patients who achieve remission but still have lower microbial diversity are more likely to experience relapse.

Biodiversity is positively correlated with ecological function, and ecological function can maintain a balanced state when the biodiversity is at a certain level for functional redundancy [81, 82]. When the balance is disrupted, the biodiversity reduces and the normal function of the ecosystem is transformed to another state, which is undesirable [83]. To determine the precise dysfunction in the intestinal ecosystem of patients with IBD, Morgan et al. studied microbial metabolism in IBD patients and healthy subjects using shotgun metagenomes. They identified major shifts in metabolic pathways, including an increase in the occurrence of oxidative stress pathways and a decrease in basic metabolism and short-chain fatty acid (SCFA) production [84]. The microbiome of patients with ileal CD is known to exhibit heightened virulence and secretion pathways [84]. One study compared the metagenome of the microbiota between children with active CD with a reduced diversity and altered stool microbial composition and healthy controls. Modules that were more abundant in the CD group included ubiquinone and lipopolysaccharide (LPS) biosynthesis, as well as the twin-arginine translocation system; sulfur reduction was also noted, whereas key processes such as fatty acid biosynthesis were overrepresented in the controls [21]. LPS can activate the toll-like receptor 4 (TLR-4) signaling pathway to induce inflammation [85]. Duboc et al. compared stool samples from IBD patients and healthy subjects to study the impact of dysbiosis in IBD on metabolism to bile acids and inflammation of the epithelium. The microbiota of IBD patients exhibited impaired deconjugation, transformation, and desulfation to bile acids, demonstrating an increase in the conjugated bile acid rates and fecal 3-OH-sulfated bile acids as well as a decrease in the secondary bile acids. The results also showed that secondary bile acids could lead to anti-inflammation activities [86].

The reduction in diversity, accompanied by alterations in the microbial structure, could induce a disturbance in normal intestinal ecological function, which is harmful to the host [84]. These phenomena suggest that a reduction in microbial diversity has a far-reaching impact on the gut ecosystem, deteriorating the gut environment and inducing intestinal inflammation. Future research should further investigate the relationship between microbial diversity and intestinal health to promote the clinical remission of intestinal inflammation via biotherapy.

## 6. Conclusion

The diversity of microbiota contributes to the stability of the intestinal ecosystem and its ecological function. A loss of diversity could initiate an inflammatory reaction and promote the development of inflammatory disease. Bacterial therapy, including FMT, probiotics, and prebiotics, has obtained curative efficacy accompanied by an improvement in diversity [87–92]. The restoration of normal microbiota diversity represents a promising prospect in curing corresponding inflammatory disease.

## Abbreviations

IBD: Inflammatory bowel disease
FMT: Fecal microbiota transplantation
UC: Ulcerative colitis
CD: Crohn's disease
TI: Terminal ileum
CDI: * Clostridium difficile* infection
rCDI: Recurrent* Clostridium difficile* infection
SCFA: Short-chain fatty acid
LPS: Lipopolysaccharide
TLR-4: Toll-like receptor 4.
